# Calpain7 impairs embryo implantation by downregulating β3-integrin expression via degradation of HOXA10

**DOI:** 10.1038/s41419-018-0317-3

**Published:** 2018-02-19

**Authors:** Qiang Yan, Chenyang Huang, Yue Jiang, Huizhi Shan, Ruiwei Jiang, Junxia Wang, Jingyu Liu, Lijun Ding, Guijun Yan, Haixiang Sun

**Affiliations:** Reproductive Medicine Center, The Affiliated Drum Tower Hospital of Nanjing University Medical School, Nanjing, 210008 People’s Republic of China

## Abstract

Endometriosis (ENDO) is a common gynecological disease that causes infertility in many women. Previous studies noted that the dysregulation of Homeo box A10 (HOXA10) in the endometrium of women with ENDO was involved in the failure of embryo implantation. However, the mechanism by which HOXA10 expression is reduced in women with ENDO is still poorly understood. Here we found that a member of the calcium (Ca^2+^)-dependent cysteine protease family calpain7 (CAPN7), negatively correlated with HOXA10, was highly expressed in the endometrium of infertile women with ENDO and was significantly downregulated during the window of embryo implantation in mice. Overexpression of CAPN7 in Ishikawa cells or in the uterus of mice inhibited embryo implantation in vitro and in vivo. In the current study, we identified a sequence rich in proline, glutamic acid, serine, and threonine (PEST sequence) that enhanced the Ca^2+^-dependent degradation of HOXA10 by CAPN7. Furthermore, the interaction between HOXA10 and CAPN7 repressed the transcriptional activity and protein stability of HOXA10. In contrast, the administration of the calpain inhibitor ALLN reversed the CAPN7-induced HOXA10 degradation. Moreover, truncation of the PEST motif in HOXA10 abolished its CAPN7-dependent proteolysis. These studies reveal a novel pattern of HOXA10 regulation via PEST sequence-mediated calpain proteolysis that was demonstrated to be reversed by a calpain inhibitor. Thus, the inhibition of CAPN7-induced HOXA10 degradation may represent a novel potential therapeutic method to improve impaired embryo implantation in women with ENDO.

## Introduction

Endometriosis (ENDO) is characterized as an estrogen-dependent^1^ disease that occurs in 5–10% of reproductive-age women^2^. ENDO is classically defined as the presence and growth of endometrial glands and the stroma outside of the uterus, mainly in the peritoneal cavity and ovary. Most women with ENDO experience chronic pelvic pain^3^ and infertility^4^. Approximately 25–50% of infertile women have ENDO and 30–50% of women with ENDO are infertile^5^. Despite extensive research, no agreement has been reached on the several mechanisms proposed to explain the association between ENDO and infertility^6^. Moreover, the mechanisms involved in ENDO infertility are complex and require further investigation.

A more recently accepted hypothesis is that the infertility experienced by some women with ENDO is due to an abnormal eutopic endometrium and implantation failure^3^. In addition, more evidence has supported the idea that aberrant gene expression in the eutopic endometrium of women with ENDO may contribute to infertility^7^. Several genes are known to be dysfunctional in the eutopic endometrium of women with ENDO, including the genes involved in the process of embryo implantation. Homeo box A10 (*HOXA10*), a key transcriptional factor considered to be a biomarker for the window of embryo implantation^8^, is proved to form dimers or trimmers through binding with TALE homodomain proteins (Pbx2 and Meis1)^9^. As cofactors, Pbx2/Mesi1 bind with HOXA10 to activate or repress the target genes expression, such as EMX2, IGFBP1, and integrin β3 (ITGB3)^10–14^. HOXA10 is expressed dynamically trough the menstrual cycle under the regulation of estrogen and progesterone, reaching high levels at the time of embryo implantation^15^. In the endometrium of women with ENDO, HOXA10 expression is significantly decreased^13, 16^. Mounting evidence indicated that reduction in HOXA10 expression induced the failure of embryo implantation^17,18]^. However, the mechanism of HOXA10 downregulation is unknown. The dysfunction of posttranscriptional regulation, such as the abnormal *HOXA10* methylation^19^ and dysregulation of miR-135a/b^20^, was reported to contribute to decrease HOXA10 expression in the endometrium of ENDO women^13, 21^. During the same time, our group elucidated that the stability of HOXA10 protein was also impaired via the abnormal interaction between p300/CBP-associated factor and HOX10 in ENDO women^22^, which led to impaired embryo implantation. Thus, the identification of new HOXA10-binding partners may provide mechanistic insights into the regulation of gene expression during embryo adhesion.

To further explore new HOXA10-binding partners, we performed a yeast two-hybrid screening to identify HOXA10-interacting proteins by using a human endometrium cDNA library. We identified calpain7 (CAPN7) as a HOXA10-interacting protein that negatively regulates HOXA10 activity in the endometrium. *CAPN7* is a member of the calpains family. In 1964, calpains were first recognized as Ca^2+^-dependent cysteine proteases and thus far 15 members have been identified^23, 24^. Calpains deficiencies and overactivation are linked to a variety of diseases and pathological consequences^25^. Due to their multifaceted nature, calpains control various irreversible signaling events and biological functions in the cell such as endothelial cell adhesion, differentiation, migration, proliferation, cell cycle control, cytoskeletal remodeling, embryonic development, and vesicular trafficking^26^. A protein with a polypeptide sequence enriched in proline (P), glutamate (E), serine (S), and threonine (T) (PEST motif) may be a target for degradation by calpains^27, 28^.

In the present study, we discovered that one of the two PEST-like sequences in HOXA10 has an essential role in determining substrate susceptibility to CAPN7 in vitro. Moreover, we demonstrated the functional significance of the CAPN7-mediated HOXA10 degradation in downregulating HOXA10 protein expression and its downstream target ITGB3 protein expression. Therefore, we hypothesized that the aberrant CAPN7 expression in the eutopic endometrium of the infertile women with ENDO may cause failure of embryo implantation via degradation of HOXA10.

## Result

### The interaction between CAPN7 and HOXA10 in Ishikawa cells

**Fig. 1:**
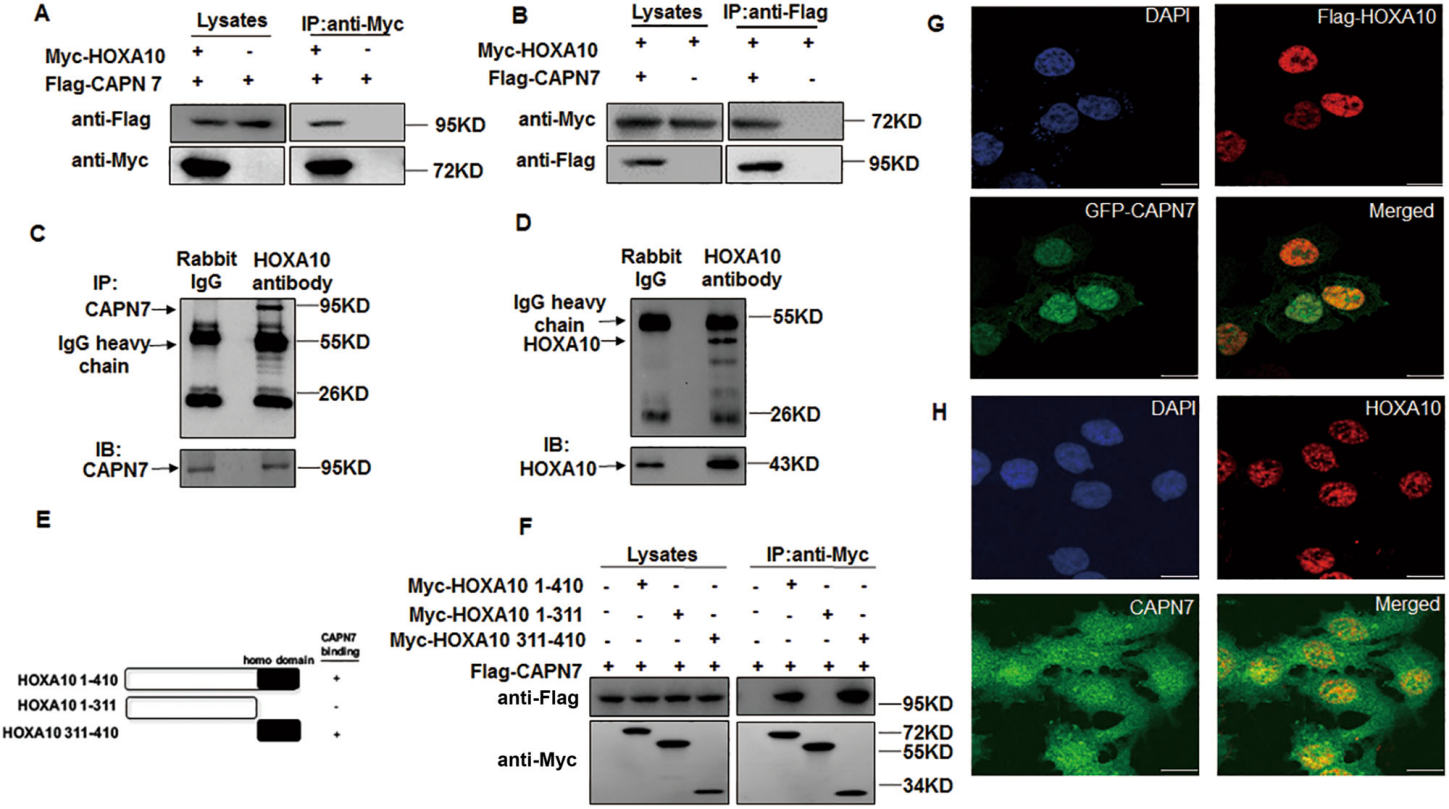
The interaction between CAPN7 and HOXA10. **a** Immunoblot analysis of whole cell lysates and anti-Myc immunoprecipitation of HEK293T cells transfected with Flag-CAPN7 alone or together with Myc-HOXA10. **b** Immunoblot analysis of whole-cell lysates and anti-Flag immunoprecipitation of HEK293T cells transfected with Myc-HOXA10 alone or together with Flag-CAPN7. **c**, **d** Immunoblot analysis of Ishikawa whole-cell lysates and anti-HOXA10 immunoprecipitation. Goat IgG was used as a negative control for the co-IP procedure. **e** Schematic representation of the HOXA10 mutants used in **f**. **f** Immunoblot analysis of whole-cell lysates and anti-Flag immunoprecipitations of HEK293T cells transfected with Flag-CAPN7 and various Myc-HOXA10 constructs. **g** Cellular localization of Flag-HOXA10 and GFP-CAPN7. Flag-HOXA10 and GFP-CAPN7 localized primarily to the nucleus and the colocalization of the two proteins was apparent in the merged image. **h** Cellular localization of endogenous HOXA10 and CAPN7 in the nuclear of Ishikawa cells

A yeast two-hybrid screen was carried out to identify novel binding partners for HOXA10. One of the novel positive clones identified was the CAPN7 550–813 AA clone. In HEK293T cells, Myc-HOXA10 specifically interacted with Flag-tagged CAPN7 (Fig. 1a,b). In addition, endogenous CAPN7 could also interact with HOXA10 proteins in Ishikawa cells (Fig. 1c,d). In the domain study, we further identified the association between CAPN7 and the C terminus (311–410 AA) of HOXA10, which contains the homeodomain (Fig. 1e,f). Furthermore, endogenous and exogenous nuclear co-localization of both proteins was observed by fluorescence confocal microscopy in Ishikawa cells (Fig. 1g,h).

### CAPN7 inhibits embryo implantation in vivo

**Fig. 2:**
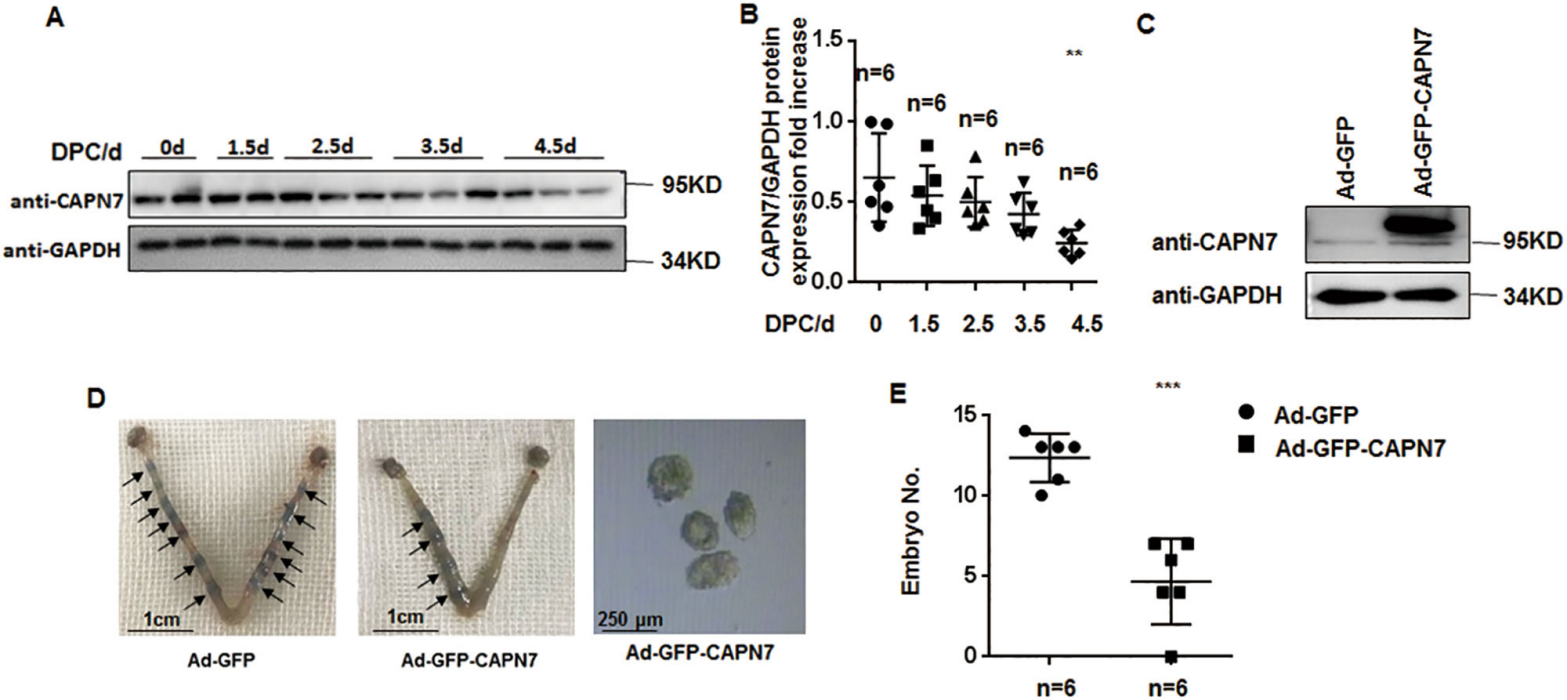
CAPN7 inhibits embryo implantation in vivo. **a** Western blot analysis of uterine CAPN7 during the peri-implantation period in mice, with the strongest decreased signal detected at 4.5 dpc, when implantation begins. **b** Protein levels were normalized to GAPDH protein expression level (*n* is shown in each column, **p < 0.01 vs control). **c** Western blotting data showing uterine CAPN7 level after injection of Ad-GFP-CAPN7 or Ad-GFP. **d** Uteri from GFP-CAPN7-enhanced and control mice at 4.5 dpc of pregnancy were collected and stained with Chicago blue dye. The uteri were washed. The non-implanted blastocysts were then counted under a light microscope. Representative images are shown. **e** The number of implanted embryos was counted (*n* is shown in each column, ***p < 0.001 compared with control)

To determine whether CAPN7 has functional consequences in terms of regulating embryo implantation, we found that CAPN7 expression was dramatically decreased on 4.5 day post coitus (dpc) in pregnant mice (*p *< 0.01) (Fig. 2a,b). Moreover, the uterine CAPN7-overexpressed mice had fewer implantation sites on 4.5 dpc than the control group (GFP-CAPN7 3.667 ± 1.282 vs green fluorescent protein (GFP)-vehicle 12.8 ± 0.4899*, p *< 0.001) (Fig. 2c,e). In addition, the average number of non-implanted blastocysts in CAPN7-overexpressed uteri was 4.17 ± 2.23, whereas we did not observe any non-implanted blastocysts in the control group (Fig. 2d), suggesting that enhanced uterine CAPN7 expression blocked embryo implantation.

### CAPN7 impairs embryo implantation via downregulating HOXA10 expression

**Fig. 3:**
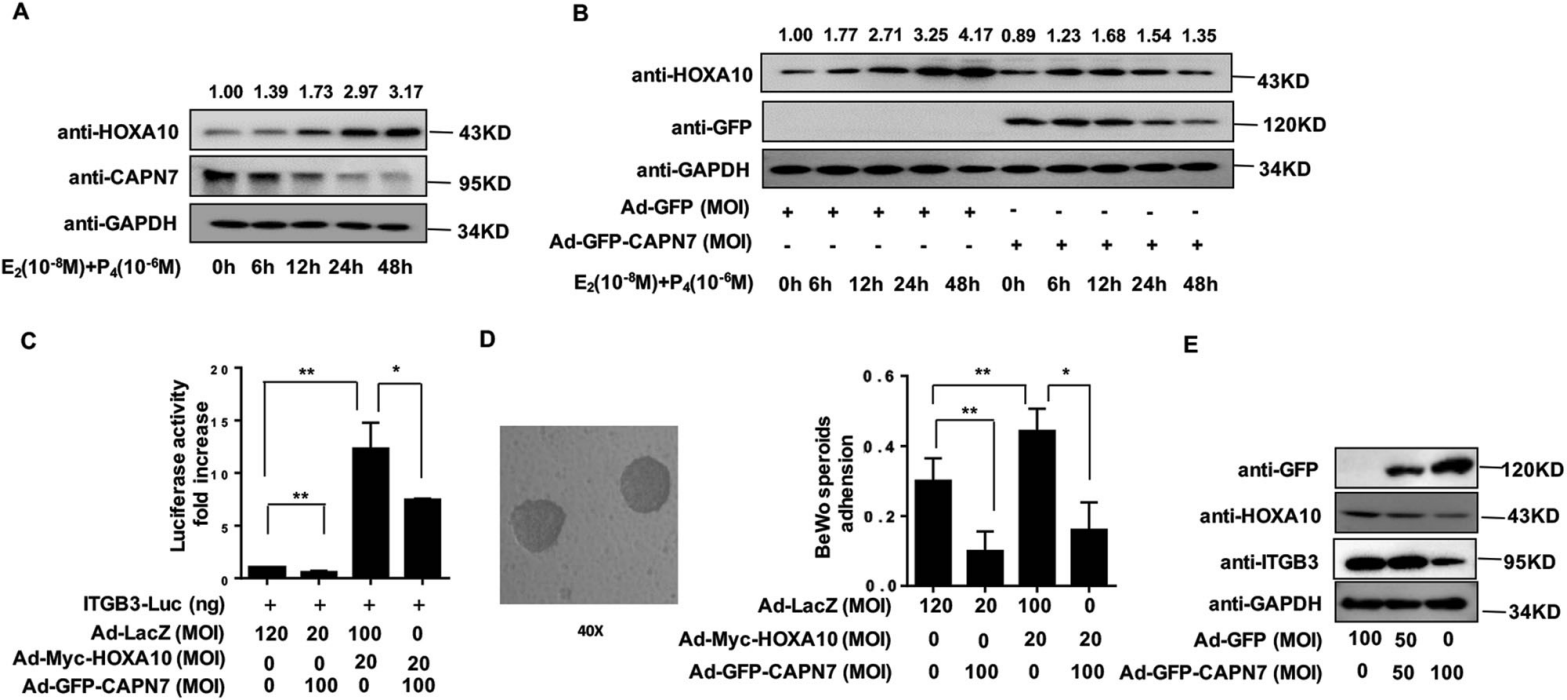
CAPN7 downregulates the expression of HOXA10 and inhibits embryo implantation. **a** Ishikawa cells were administered estrogen (10^–^^8^ M) and progesterone (10^–6^ M) at different times, as indicated. Whole-cell lysates were analyzed by western blot analysis with the indicated antibodies. The values of the density were marked above the band. **b** The Ishikawa cells transduced with Ad-GFP-CAPN7 (100 MOI)were treated with estrogen (E) (10^–8^ M) and progesterone (P) (10^–6^ M) at different times, as indicated. Whole-cell lysates were analyzed by western blot analysis with the indicated antibodies. The values of the density were marked above the band. **c** Ishikawa cells were transfected with ITGB3-Luc, Ad -Myc-HOXA10 (20 MOI), and Ad-GFP-CAPN7 (100 MOI), as indicated. After 48 h, the luciferase activities were measured and are presented as fold induction. Values represent the mean ± SEM (*n* = 3), *p < 0.05, **p < 0.01. **d** BeWo spheroids (150–200 μm diameter) were attached to Ishikawa cells after 2 h of co-culture. Adhesion experiments with BeWo spheroids attached to the Ishikawa cell monolayer. The data are the average of three independent experiments. An ANOVA test was used to compare the percentage of the attached spheroids with each treatment in comparison to the control. The error bars indicate SD of three independent experiments. Values represent the mean ± SEM (*n* = 3), *p < 0.05, **p < 0.01. **e** Ishikawa cells were transduced with Ad-GFP or Ad-GFP-CAPN7 (100 MOI) as indicated. Western blot analysis was performed with the indicated antibodies

As shown in Fig. 3a, the endogenous CAPN7 protein levels were decreased in Ishikawa cells treated with estrogen and progesterone in a time-dependent manner, whereas the HOXA10 expression was increased. In addition, enhanced CAPN7 expression could repress estrogen- and progesterone-induced HOXA10 expression (Fig. 3b). As the interaction between CAPN7 and HOXA10 was confirmed, we further investigated whether CAPN7 modulated HOXA10 function. Luciferase reporter assay results demonstrated that ITGB3 activity, which is transcriptionally regulated by HOXA10, was significantly reduced by ~ 50% in CAPN7-enhanced Ishikawa cells (Fig. 3c, *p *< 0.05). In addition, an appropriate trophoblast-epithelial cell interaction model confirmed that ectopic CAPN7 expression repressed the adhesion of BeWo cell spheroids to Ishikawa cells that was induced by HOXA10 overexpression (Fig. 3d, *p *< 0.05). In addition, we also found that enhanced CAPN7 expression decreased the expression levels of HOXA10 and ITGB3 protein expression (Fig. 3e). However, Fig. S1A and B showed that CAPN7 had no effect on HOXA10 mRNA level in Ishikawa cells, suggesting that CAPN7 decreased HOXA10 expression via posttranslational modification.

### CAPN7-induced HOXA10 degradation results in decreasing protein stability and DNA-binding ability

**Fig. 4:**
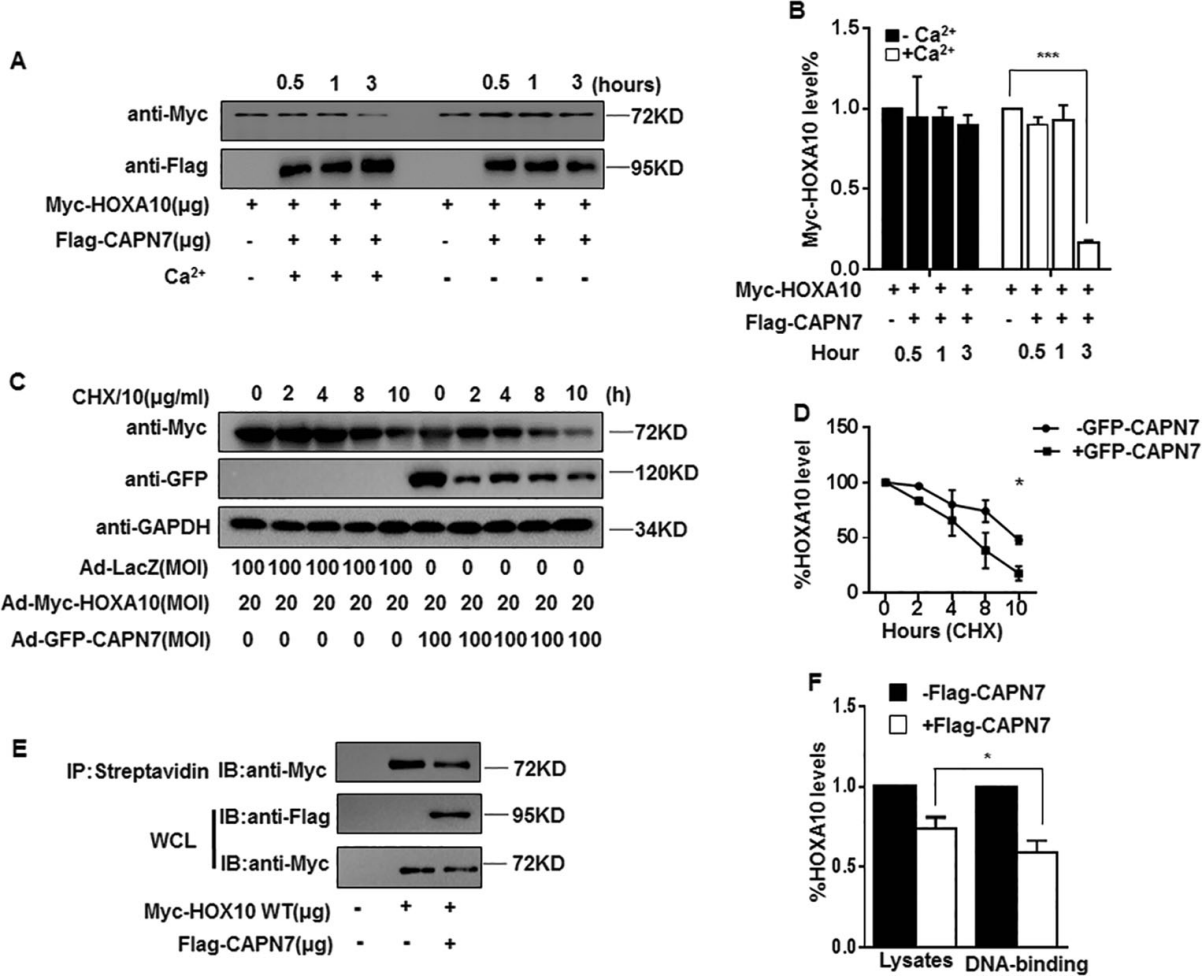
CAPN7-mediated HOXA10 degradation impairs the protein stability and DNA-binding ability of HOXA10. **a** CAPN7 degrades HOXA10 in vitro. In the presence of Ca^2+^ (3 mM) for 3 h at 37 °C. Lysates were analyzed by western blotting with the indicated antibodies. **b** The intensities of Myc-HOXA10 signals were quantified from the different groups and normalized to the control group (hour = 0 h). Values represent the mean ± SEM (*n* = 3), ***p < 0.001 vs hour = 0 group. **c** Ishikawa cells were transduced with Ad-GFP-CAPN7 (100 MOI) and Ad-Myc-HOXA10 or with Ad-Myc-HOXA10 and Ad-LacZ. At 24 h posttransduction, cycloheximide (CHX, 10 μg/ml) was added to the cell cultures, total proteins were isolated, and the levels of CAPN7 and HOXA10 were examined by western blot analysis at the indicated times. **d** The intensities of HOXA10 signals were quantified from three independent experiments and normalized to GAPDH. The results are expressed as the percentage relative to the levels observed at time 0. The error bars indicate SD of three independent experiments. At time 10 h, *p < 0.05 vs control. **e** In a biotin-labeled DNA pull-down assay, cell extracts from HEK293T cells treated with the indicated plasmid were incubated with biotinylated DNA probe. After incubating with streptavidin sepharose beads, the extracts were subjected to western blot analysis. **f** The intensities of HOXA10 signals were quantified from three independent experiments and normalized to GAPDH. *p < 0.05

As calpains degrade downstream proteins by targeting the PEST motif, we identified two conserved potential PEST sequences in HOXA10 by using the program ePESTfind (e*mboss.bioinformatics.nl*) (Figs. S2A and 2B; PEST1 score, + 9.37; PEST2 score, + 11.45; PEST scores > + 5 are considered significant), suggesting that HOXA10 may be degraded by CAPN7. Indeed, CAPN7 effectively degraded ~ 80% of total HOXA10 protein in a Ca^2+^-dependent manner (Fig. 4a,b), resulting in a reduced half-life of HOXA10 protein (~ 5 h, *p *< 0.05) (Fig. 4c,d) and suppressed HOXA10 activation and thus its regulation of downstream genes expression (Fig. 4e,f).

### The PEST motif of HOXA10 is critical for CAPN7-mediated degradation

**Fig. 5:**
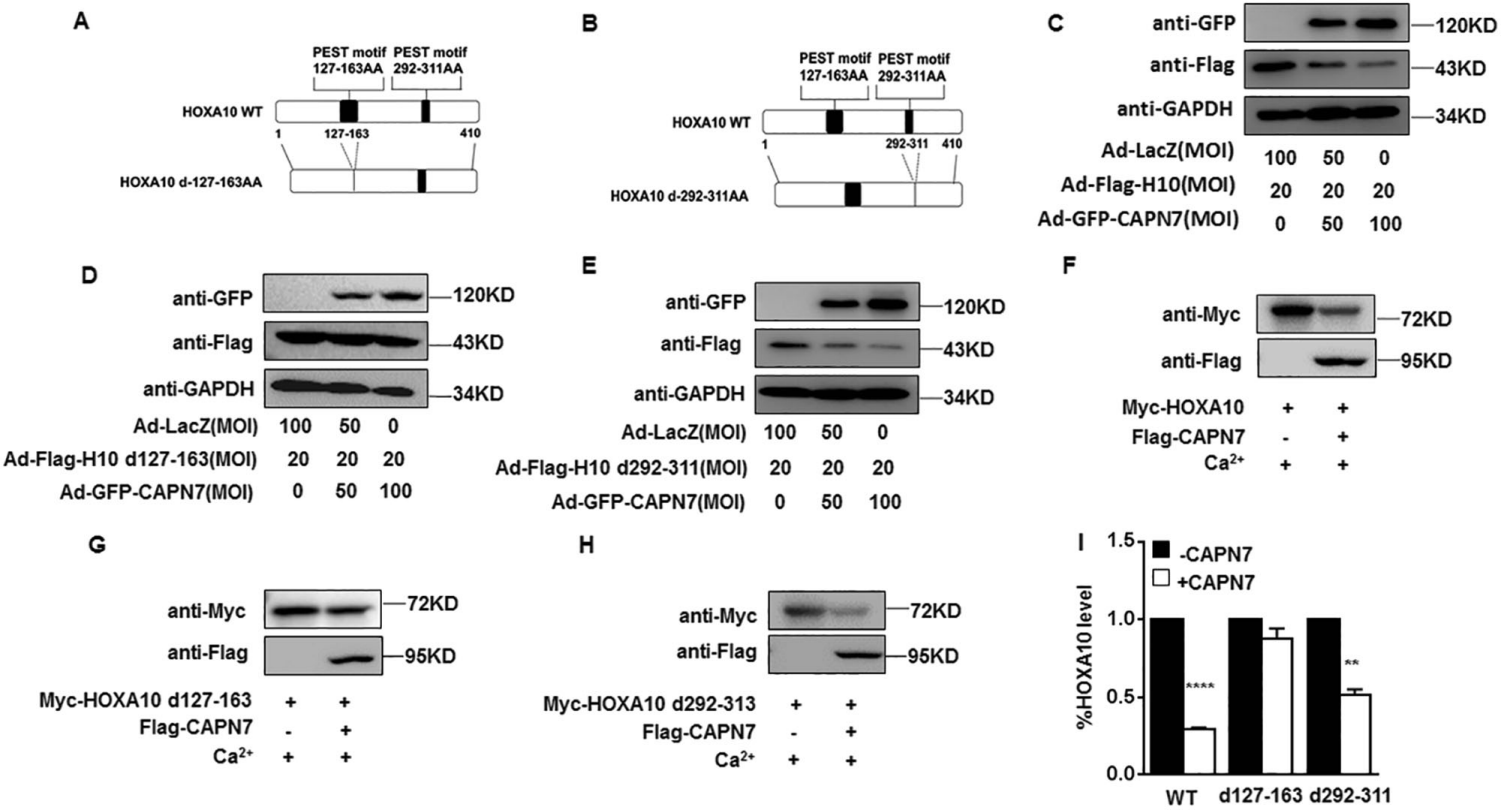
PEST motif 127-163 is critical for CAPN7-mediated HOXA10 degradation **a**, **b** Schematic model of generating the HOXA10 WT and HOXA10 PEST deletion mutant (HOXA10–dPEST) constructs. **c** Ishikawa cells transfected with Ad-GFP-CAPN7 and Ad-Flag-HOXA10 WT. Whole-cell lysates were detected by western blot analysis with the indicated antibodies. **d** Ishikawa cells transfected with Ad-GFP-CAPN7 and Ad-Flag-HOXA10 d127-163. Whole-cell lysates were detected by western blot analysis with the indicated antibodies. **e** Ishikawa cells transfected with Ad-GFP-CAPN7 and Ad-Flag-HOXA10 d292-311. Whole-cell lysates were detected by western blot analysis with the indicated antibodies. **f** CAPN7 degraded HOXA10 in vitro. **g** In vitro degradation assay, indicating that CAPN7 had a slight effect on HOXA10 d127-163. **h** Flag-CAPN7 degraded HOXA10 d292-311 in vitro. **i** The intensities of degraded HOXA10 signals were quantified from three independent experiments and normalized to the control group. Values represent the mean ± SEM (*n* = 3), ****p < 0.0001; **p < 0.01

As HOXA10 had two predicted PEST motifs at amino acids 127–163 and 292–311, we generated PEST motif-deletion-expressing constructs, namely HOXA10-d127-163 and HOXA10-d292-311 (Fig. 5a,b). We observed that enhanced GFP-CAPN7 expression in Ishikawa cells significantly downregulated the expression of Flag-HOXA10 WT and Flag-HOXA10 d292-311 but not Flag-HOXA10 d127-163 (Fig. 5c,d,e). In addition, exogenous Flag-CAPN7 promoted the degradation of Myc-tagged HOXA10 WT and Myc-HOXA10 d292-311 rather than Myc-HOXA10 d127-163 in vitro (Fig. 5f,g,h). Above all, the PEST motif 127–163 of HOXA10 was particularly important for CAPN7-dependent proteolysis.

### A calpain inhibitor reverses HOXA10 degradation and promotes embryo implantation

**Fig. 6:**
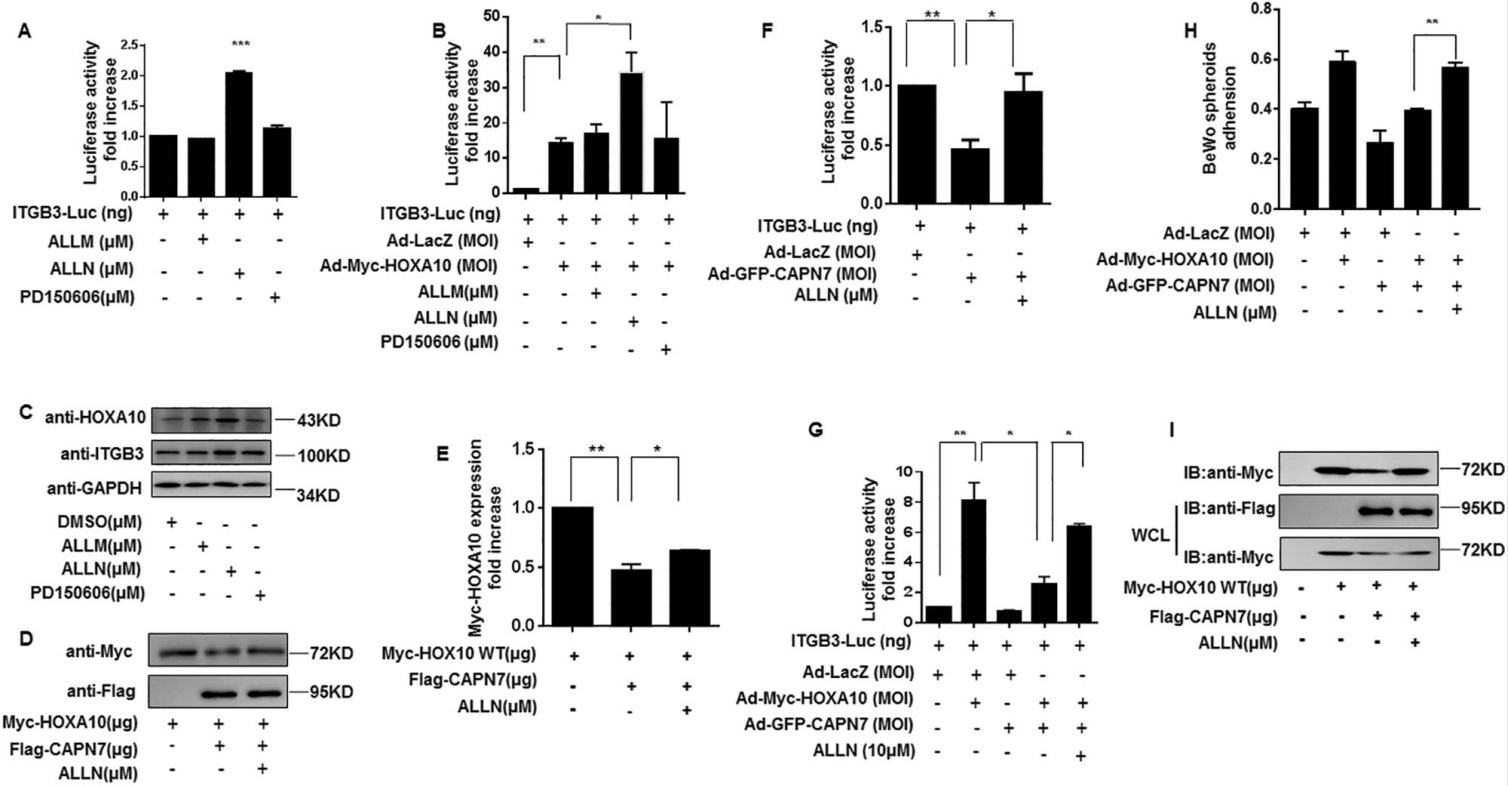
ALLN inhibits the activity of CAPN7. **a** Ishikawa cells were transfected with ITGB3-Luc and were treated with ALLM, ALLN, and PD150606 (10 μM). After 48 h, the luciferase activities were measured and are presented as fold induction. Values represent the mean ± SEM (*n* = 3). ***p < 0.001. **b** Ishikawa cells were transfected with ITGB3-Luc and Myc-HOXA10, and were treated with ALLM, ALLN, and PD150606 (10 μM). After 48 h, the luciferase activities were measured and are presented as fold induction. Values represent the mean ± SEM (*n* = 3). *p < 0.05, **p < 0.01. **c** Western blot analysis of whole-cell lysates from Ishikawa cells treated with ALLM, ALLN, and PD150606 (10 μM), as indicated. The membrane was probed with the indicated antibodies. **d** Calpain inhibitor ALLN inhibited the degradation of HOXA10 by CAPN7. **e** The intensities of HOXA10 signals were quantified from three independent experiments and normalized to the control group. *p < 0.05, **p < 0.01. **f** ALLN inhibited CAPN7-induced downregulation of transcriptional activity. Values represent the mean ± SEM (*n* = 3); *p < 0.05, **p < 0.01. **g** ALLN reversed GFP-CAPN7-downregulated HOXA10 transcriptional activity. Values represent the mean ± SEM (*n* = 3); *p < 0.05, **p < 0.01. **h** ALLN suppressed CAPN7-induced BeWo adhesion. Values represent the mean ± SEM (*n* = 3); **p < 0.01. **i** ALLN rescued the CAPN7-downregulated HOXA10 DNA-binding ability

Three common calpain inhibitors (ALLM, ALLN, and PD150606) at 10 μM were chosen to detect the effect of endogenous CAPN7 on HOXA10 expression in Ishikawa cells. Fig. 6a,b showed that only ALLN resulted in an increase in HOXA10-induced ITGB3-Luc activity (*p < *0.05). In addition, ALLN further contributed to the induction of HOXA10 expression and the protein expression levels of its downstream target ITGB3 (Fig. 6c). The selected inhibitor ALLN was used in the following studies. In Fig. 6d,e, ALLN blocked HOXA10 degradation, which led to increased transcriptional activity (Fig. 6f,g), improved BeWo spheroid adhesion (Fig. 6h) and induced DNA-binding ability of HOXA10 (Fig. 6i). These results demonstrated that ALLN is a potential CAPN7 inhibitor.

### Aberrant CAPN7 expression in the eutopic endometrium of women with ENDO

**Fig. 7:**
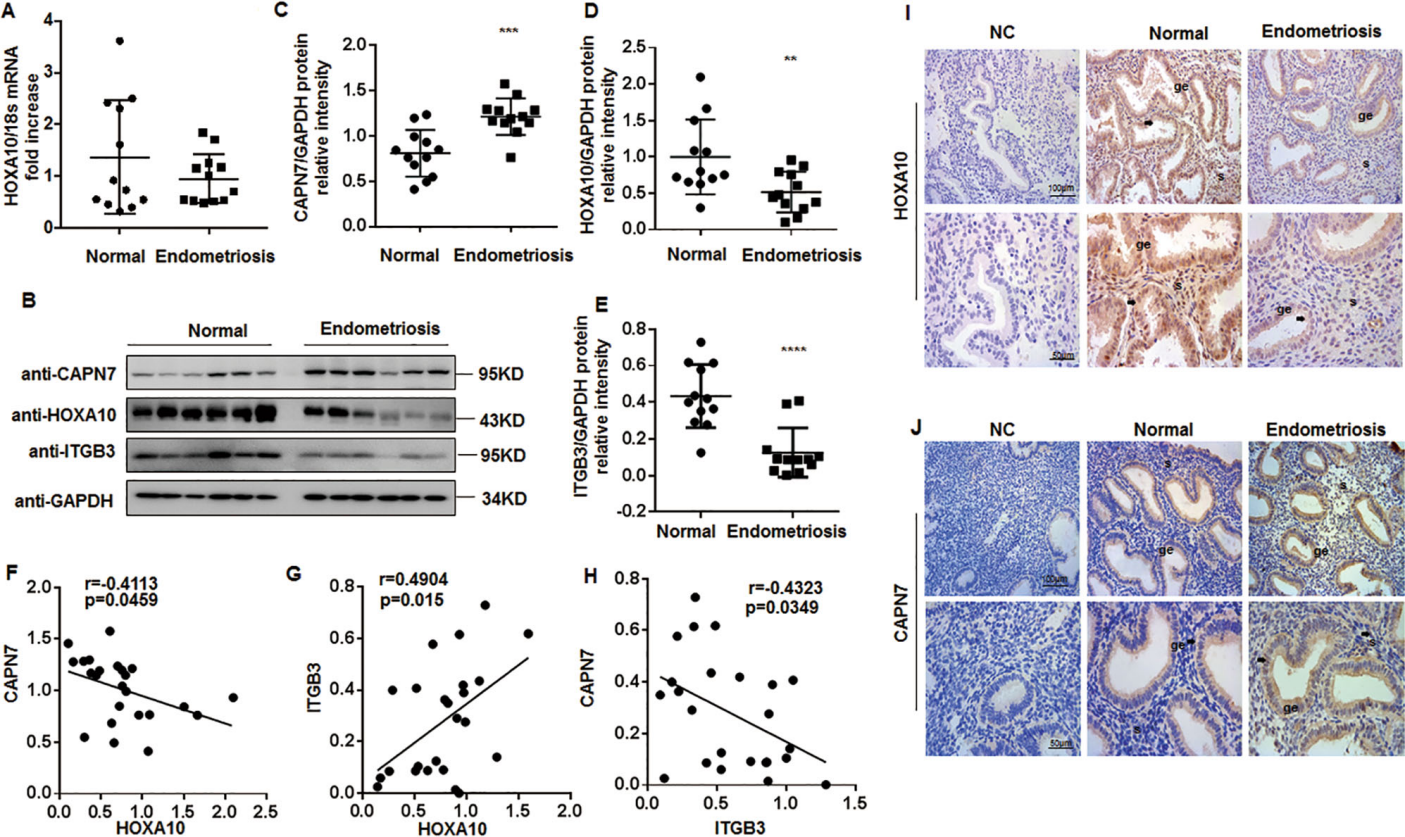
Aberrant overexpression of CAPN7 in the endometrium of women with ENDO. **a** Timed mid-secretory endometrial biopsies from healthy controls (*n* = 12) and infertile women with ENDO (*n* = 12) were analyzed for the mRNA expression of HOXA10 by qRT-PCR analysis. **b** Timed mid-secretory endometrial biopsies from healthy controls (*n* = 12) and infertile women with ENDO (*n* = 12) were analyzed for HOXA10 and CAPN7 protein expression using western blot analysis. **c** The intensities of CAPN7 signals were quantified from the 12 samples and normalized to GAPDH. ***p < 0.001. **d** The intensities of HOXA10 signals were quantified from the 12 samples and normalized to GAPDH. **p < 0.01 vs control. **e** The intensities of ITGB3 signals were quantified from the 12 samples and normalized to GAPDH. ****p < 0.0001 vs control. **f** Correlation between CAPN7 and HOXA10 expression in endometrial samples of women with or without ENDO (*r *= -0.4113, *p *= 0.0459). **g** Correlation between ITGB3 and HOXA10 expression in endometrial samples of women with or without ENDO (*r *= 0.4904,* p *= 0.015). **h** Correlation between CAPN7 and ITGB3 expression in endometrial samples of women with or without ENDO (*r *= -0.4323, *p *= 0.0349). **i** Timed mid-secretory endometrial biopsies from healthy control (*n* = 3) and infertile women with ENDO (*n* = 3) were analyzed using immunohistochemistry (IHC). Goat IgG was used as a negative control. Arrows show the increased HOXA10 conjugates in the glandular epithelium. **j** Timed mid-secretory endometrial biopsies from healthy control (*n* = 3) and infertile women with ENDO (*n* = 3) were analyzed using immunohistochemistry (IHC). Rabbit IgG was used as a negative control. Arrows show the increased CAPN7 conjugates in the glandular epithelium

In the endometrium from all volunteers shown in Table 1, the protein level of HOXA10 was decreased only in ENDO women, but the mRNA level was similar (Fig. 7a–c). Correspondingly, the protein expression of HOXA10 target ITGB3 decreased significantly (Fig. 7b,d). Conversely, the expression of CAPN7 in the endometrium of infertile ENDO women was ~ 1.5-fold compared with the control group (*p *< 0.001) (Fig. 7b,e). In addition, as shown in Fig. 7f,h, both of the protein levels of HOXA10 and ITGB3 were moderately negatively correlated with CAPN7 respectively (HOXA10: *r *= −0.4113, *p *= 0.0459; ITGB3: r = −0.4323, *p *= 0.0349). Furthermore, the immunolocalization analysis showed a higher level of CAPN7 protein in the endometrium of ENDO women than that in fertile controls, especially in glandular epithelial cells. **(**Fig. 7f). These results suggested that CAPN7 decreased HOXA10 expression in ENDO women, resulting in impaired embryo implantation.

**Table 1:** Demographic details of the participants in the study of endometrial CAPN7 and HOXA10 expression

Disease	Normal (*n* = 12)	EMT (*n* = 12)	*P*
Age (years)	28.6 ± 1.0	28.9 ± 0.8	NS
Body mass index (kg/m^2^)	21.3 ± 0.6	21.8 ± 0.9	NS
Menstrual cycle (days)	29.4 ± 0.3	29.1 ± 0.5	NS

The data are presented as the mean ± SD unless otherwise indicated. *P *< 0.05 was considered significant.

## Discussion

The association between ENDO and infertility is well established^13^; however, the exact mechanisms by which ENDO causes implantation failure are unclear. This study is the first to demonstrate that the aberrantly increased expression of a physiological binding partner of HOXA10 CAPN7 in ENDO women suppresses embryo implantation.

Embryo implantation is a dynamic process that includes apposition, adhesion, attachment, and penetration^29^. The transcription factor HOXA10 is strongly expressed from 1.5 to 4.5 dpc in the mouse uterus and has a critical role in embryo implantation. A previous study found that CAPN7 promotes the migration and invasion of human endometrial stromal cells (HESCs) during ENDO pathogenesis by interacting with a matrix metalloproteinase 2 transcription factor AP-2α^30^. As a degradation regulator of HOXA10, we found in our current study that CAPN7 expression is continuously decreased in ENDO women and is ultimately reduced to a minimal level by 4.5 dpc in mouse. Moreover, expression of exogenous CAPN7 repressed HOXA10 protein stability and DNA-binding ability, which resulted in embryo implantation failure, suggesting that aberrant overexpression of CAPN7 in ENDO endometrium might impair embryo implantation. Furthermore, previous studies indicated the involvement of other non-classical calpains in ENDO, such as CAPN5 and CAPN6 (ref. ^31, 32^). However, there was no difference in the expression levels of classical calpains, such as CAPN1 and CAPN2, in the endometrium of ENDO women (data not shown). These results suggest that CAPN7 plays an important role in ENDO associated with embryo implantation failure.

As a protease, calpains could degrade several transcription factors in many diseases, including Wnt^33, 34^, STAT3/STAT5 (ref. ^35^), and Foxo-signaling pathways^36^, all of which contribute to the process of embryo implantation^37–41^. Together with the PEST motif prediction, we demonstrated that HOXA10, as an endometrial receptivity marker, was degraded via CAPN7 targeting the PEST motif (127–163 AA). Previous studies revealed that the protease MMP9 cleaves steroid receptor coactivator-1 into a 70 kDa C-terminal isoform in endometriotic tissue, which promotes pathogenic progression of ENDO^42^. Meanwhile, in our study, because of the lack of suitable antibodies available for the detection of endogenous and exogenous HOXA10 expression, we did not find the specific fragments left after the degradation. Future studies are required to confirm whether the mechanism uncovered in this study reflects the regulation of the degraded HOXA10 isoform in embryo implantation.

Recent studies indicated that the activation of non-classical calpains is Ca^2+^-independent^43, 44^. However, we found that CAPN7 functioned as a Ca^2+^-dependent cysteine proteases and the C2 domain-like (C2L) domain had important roles in CAPN7 activity. Within the CAPN7 sequence, the C2L domain follows the CysPc domain (PC1 and PC2 domains), which contains two unique Ca^2+^-binding sites (CBS-1 and -2, respectively)^45, 46^. Upon binding Ca^2+^, the PC1 and PC2 domains rotate toward each other to form an active binding site for HOXA10. Thus, the presence of Ca^2+^ enables CAPN7 to degrade more HOXA10. Despite the degradation of total HOXA10 protein, CAPN7 can also decrease the DNA-binding ability of HOXA10. We speculated that there are several possible mechanisms in which the CAPN7–HOXA10 interaction might decrease the DNA-binding affinity of HOXA10. Previous studies found that the HOXA10 124–164 domain recruited CBP to the ITGB3 promoter. This domain was homologous to PQ domains that mediate protein–protein interactions between other transcriptional activator proteins. Consistent with this, our additional studies showed that deletion of the HOXA10 127–163 domain significantly decreased its transcriptional activity (data not shown). Accordingly, CAPN7 targeted the 127–163 region, which contains the PQ-like domain, resulting in the decreased HOXA10 DNA-binding affinity. However, the precise mechanism requires further investigation. In fact, apart from ITGB3, the expression of EMX2, a regulator of endometrial proliferation^47^, was abnormally increased due to the altered HOXA10, which was potentially to affect the endometrium receptivity in the EDNO^48, 49^. The influence of HOXA10 degradation on the form of heterodimers, an enhancer of EMX2, leading to the altered EMX2 expression needs further studies. This may provide new ideas for the treatment of embryo implantation failure in ENDO.

In the present study, ALLN was identified as a potential CAPN7 inhibitor that could rescue impaired embryo implantation via reversing CAPN7-induced degradation of HOXA10. In the calpain family, calpastatin is an endogenous inhibitor^50^. As calpastatin is able to inhibit calpains via binding domain II and domain IV or VI, which are absent in atypical calpains, we did not choose this natural inhibitor. Initially, various small molecule inhibitors were classified as active site-directed and allosteric effectors. Allosteric calpain inhibitors are most likely to interact with other sites (allosteric sites) that are involved in catalysis and activation. PD150606, one such allosteric inhibitor, regulates calpain activity via binding the PEF-hand domain, which CAPN7 lacks^23^. As a result, PD150606 had no effect on CAPN7 activity. Both ALLM and ALLN are active site effectors^23^; however, only ALLN was able to regulate CAPN7 activity. The mechanism by which ALLN affects CAPN7 activity should be further explored.

In conclusion, the present study is the first to report that human CAPN7 protein interacts with HOXA10, and that this interaction leads to the degradation of HOXA10. In addition, the aberrantly overexpressed CAPN7 in ENDO endometrium is a critical pathogenic factor for embryo implantation failure. These observations will pave the way toward further research on the regulation of CAPN7 function and provide a potential therapeutic method for ENDO-associated infertility.

## Materials and methods

### Patients and sample collection

Normal endometrium samples were collected from 15 healthy fertile women aged 23–35 years, who had no evidence of endometrial abnormalities, pelvic ENDO, or adenomyosis. All samples were collected with the informed consent of the patients and approval from the ethics committee was obtained for this study. During the mid-secretory phase of menstruation, 8–9 days after ovulation, endometrium tissue samples were collected. In contrast, eutopic endometrium tissue samples were simultaneously collected from 15 age-matched ENDO patients, who were laparoscopically diagnosed with ENDO. The mean (± SD) ages of subjects in each group were 29.0 (± 0.8) years (ENDO) and 28.4 (± 0.8) years (fertile control). There were no significant differences between the groups in age, body mass index, and phase cycle. Endometrial biopsy was performed under an approved Human Investigations Committee protocol. All patients enrolled in the study had regular menstruation and had no history of hormone treatment before surgery. All the patients signed an informed consent form before the operation and this study was approved by the Drum Tower Hospital Research and Ethics Committee.

### Cell culture, steroid hormones, and inhibitors

Ishikawa cells, HEK293T cells, and BeWo cells were maintained at 37 °C in an atmosphere of 5% CO_2_/95% air in DMEM supplemented with 10% (v/v) fetal bovine serum (Gibco BRL/Invitrogen, Carlsbad, CA, USA) and 1% penicillin/streptomycin (HyClone Laboratories, South Logan, UT, USA). Cells were cultured for the indicated durations under the treatments described in each the figure legend section. Treatments included 17β-estradiol (E, 10^−8^ M), progesterone (P, 10^−6^ M), and calpain inhibitors (ALLM, ALLN, and PD150606; 10 μM) (Sigma-Aldrich, St Louis, MO, USA).

### Co-immunoprecipitation and western blot analysis

HEK293T cells were transiently co-transfected with the indicated plasmids. At 48 h post transfection, the cells were lysed in whole-cell lysis buffer (50 mM Tris-HCl pH 7.6, 150 mM NaCl, and 1.0% NP-40) containing a protease inhibitor cocktail (Sigma-Aldrich). Next, 500 μg of cell lysates were incubated with protein A/G PLUS-agarose beads (Abmart, Shanghai, China) 4 °C for 2 h. Then, the lysates were incubated with 30 μl of Myc-conjugated antibody (Sigma-Aldrich) or 30 μl of anti-Flag M2 beads (Sigma-Aldrich) at 4 °C overnight with constant shaking. The samples were resolved by SDS-polyacrylamide gel electrophoresis (PAGE), transferred onto polyvinylidene difluoride membranes (Millipore, Billerica, USA), and analyzed by western blot analysis using an anti-Flag M2 monoclonal antibody (Sigma-Aldrich) and an anti-Myc horseradish peroxidase-conjugated antibody (Thermo Scientific, MA, USA) antibodies.

For immunoprecipitation assays in Ishikawa cells, 800 μg of protein extract were incubated with protein A/G PLUS-agarose beads (Abmart) for 2 h. After centrifuging (9,000 r.p.m., 1 min), the lysates were incubated with HOXA10 antibody (Santa Cruz Biotechnology, Dallas, TX, USA) and purified goat IgG (Invitrogen, Carlsbad, CA, USA) at 4 °C overnight. Then, the lysates were incubated with Protein G-agarose beads (Roche, Mannheim, Germany). The beads were washed three times after 2 h. Other antibodies applied in this study were CAPN7 (1 : 1,000; Santa Cruz Biotechnology, CA, USA), ITGB3 (1 : 1,000; Abgent), GFP (1 : 1,000; Bioworld Technology, MN, USA), and GAPDH (1 : 10,000; Bioworld Technology).

### RNA isolation and quantitative real-time PCR

Total RNA was extracted from cells or endometrium tissue using TRIzol reagent (Life Technologies, NY, USA). Subsequently, 1 μg of RNA was reverse-transcribed into cDNA using the PrimeScript RT reagent kit (BIO-RAD, Hercules, CA, USA), according to the manufacturer’s instructions. Quantitative real-time PCR was conducted on a MyiQ Single-Color Real-Time PCR Detection System (BIO-RAD). The HOXA10 and CAPN7 mRNA expression levels were normalized to 18S with the 2^–^^△△CT^ method. The following primer sequences were used for the indicated genes: HOXA10: 5′-AGGTGGACGCTGCGGCTAATCTCTA-3′ and 5′-GCCCCTTCCGAGAGCAGCAAAG-3′; CAPN7: 5′-ATGGTGTCCCAAGAAAGGTG-3′ and 5′-TGGTATCCAGCCAGTCAGTG-3′; 18S rRNA: 5′-CGGCTACCACATCCAAGGAA and CTGGAATTACCGCGGCT-3′.

### Immunofluorescence staining

Ishikawa cells (10^5^/well) were washed with phosphate-buffered saline (PBS) and fixed with 4% paraformaldehyde (w/v) for 20 min at room temperature. After washing three times with PBS for 5 min, the fixed coverslips were permeabilized in PBS with 0.1% Triton X-100 for 5 min at room temperature. Nonspecific sites were blocked with 3% bovine serum albumin in PBS for 1 h at 37 °C. The cells were incubated with an anti-HOXA10 polyclonal antibody (1 : 100; Santa Cruz Biotechnology) and an anti-CAPN7 monoclonal antibody (1 : 100; Novus, Littleton, Colorado, USA) at 4 °C overnight. After washing with PBS (three times), the coverslips were further incubated with Alexa Fluor 594-conjugated donkey anti-goat IgG (1 : 200, Invitrogen) and Alexa Fluor 488-conjugated goat anti-rabbit IgG (1 : 200, Invitrogen). Nuclei were stained with 4',6-Diamidino-2-phenylindole dihydrochloride (DAPI) (Sigma-Aldrich). Finally, images were captured by fluorescence confocal microscopy (Olympus, FV10i).

### Attachment assay of BeWo spheroids to Ishikawa cells

Ishikawa cells were seeded into a 24-well plate and transfected with the indicated adenovirus. Then, BeWo cells were detached with 0.25% trypsin (Gibco BRL/Invitrogen, Carlsbad, CA, USA) after reaching 80% confluence. The BeWo cell suspension was placed in the 35 mm^2^ dishes coated with an anti-adhesive polymer, poly-2-hydroxyethyl methacrylate (Sigma), to induce the formation of BeWo spheroids that were 150–200 μm in diameter after 48 h of culture. The spheroids were then transferred onto a confluent monolayer of Ishikawa cells. After incubation at 37 °C for 2 h, the unattached spheroids were removed by washing with PBS. The attachment rate was expressed as a percentage of the number of attached spheroids divided by the total number of spheroids added to the Ishikawa cells. Representative images are shown.

### Transfection and luciferase assays

The pGL3-basic luciferase reporter plasmid loaded with the ITGβ3 promoter was constructed as previously described^22^. HEK293T cells or Ishikawa cells were seeded into a 12-well plate, using Lipofectamine 2000 (Invitrogen, Tokyo, Japan); cells were transfected with the ITGB3-Luc plasmid and *Renilla* luciferase along with expression plasmids as indicated. At 48 h post transfection, the cells were collected. Promega (Madison, WI, USA) Dual-Luciferase Reporter Assay System was used to measure the luciferase activity according to the manufacturer’s protocol. The transfection efficiency was normalized to the activity of co-transfected *Renilla* luciferase.

### Immunohistochemistry

Human endometrium tissue sections slide (5 μm) were deparaffinized in xylene and ethanol. Endogenous peroxidase was removed with 3% H_2_O_2_ incubation for 10 min. Slides were blocked with 1.5% normal goat or rabbit blocking serum for 45 min at room temperature and the sections were incubated overnight at 4 °C with primary anti-CAPN7 antibody (1 : 200; Novus) and anti-HOXA10 (1 : 50; Abcam, Cambridge, CA, USA). After washing with PBS, the sections were incubated with a goat anti-rabbit secondary antibody at 37 °C for 30 min. Finally, the sections were stained with 3, 3′-diaminobenzidine and counterstained with hematoxylin. Nonspecific rabbit IgG and goat IgG were used as negative control, and were stained alongside the experimental sections. Nonspecific staining was not detected in the controls.

### Immunoaffinity purification of Flag-CAPN7

Forty-eight hours post transfection with the expression vector Flag-CAPN7 using Lipofectamine 2000 reagent (Invitrogen), HEK293T cells were collected with lysis buffer (20 mM Hepes⁄NaOH, pH 7.4, 150 mM NaCl, 1 mM dithiothreitol (DTT), 1 mM Pefabloc, 0.1% Triton X-100). In each sample, equal quantities were incubated with protein A/G PLUS-agarose beads (Abmart) for 2 h, followed by an incubation with 30 μl of anti-Flag M2 affinity gel (Sigma-Aldrich) overnight at 4 °C. The beads were washed five times with lysis buffer and the supernatant were eluted with 50 μl of 3 × FLAG peptide (0.1 mg/ml) (Sigma-Aldrich). Elutes were immediately used for degradation assays.

### In vitro degradation reactions

HEK293T cells were transfected with the expression vectors Myc-HOXA10, Myc-HOXA10-d127-163AA, or Myc-HOXA10-d292-311AA using Lipofectamine 2000 reagent (Invitrogen). At 48 h post transfection, the cells were collected and lysed in lysis buffer (20 mM Hepes⁄NaOH pH 7.4, 150 mM NaCl, 1 mM DTT, 1 mM Pefabloc, 0.1% Triton X-100). Then, lysates were incubated with Myc-beads (Sigma-Aldrich) overnight at 4 °C with gentle mixing. The beads were washed five times with lysis buffer and once with the buffer without detergent to eliminate residual 0.1% Triton X-100. Then, the Myc-beads were recovered by low-speed centrifugation (9,000 r.p.m.) for 1 min and incubated in the presence or absence of equal quantities of eluted Flag-CAPN7 protein plus 3 mM CaCl_2_ at 37 °C for 3 h. The reaction was stopped by adding 2 × SDS sample buffer and boiling at 95 °C for 3 min.

### DNA pull-down assay

The double-stranded oligonucleotide DNA probes with biotin were synthesized by Sangon Biotech (Shanghai, China). The primers contained triple repeats of HOXA10 binding sites (TTAT) and were biotinylated at the 5′-end. The primer sequences are as follows: forward: biotin-5′- GGGGGGCTTATAATGTTATTTTTAGTTTACA-3′; reverse: 5′-GTAAGAACCTGTAAACTAAAAATAACATTATAAGCCCCCC-3′. Ishikawa cells were transfected with the expression plasmids Myc-HOXA10 alone or together with Flag-CAPN7. pCS2-Myc and pCMV-Flag were transfected as the control plasmids. After 48 h, the cells were collected with lysis buffer. Next, 500 μg cell preclear extracts were incubated with biotinylated DNA probe for 5 h and then the mix was immobilized on streptavidin agarose (Sigma-Aldrich) in binding buffer (10 mM Tris pH 8.0, 150 mM NaCl, 0.5% (v/v) Triton X-100, 0.5 mM DTT, 0.5 mM EDTA, 10% (v/v) glycerol, 20 μg/ml poly [dI-dC], and protease inhibitor cocktail for another 4 h at 4 °C. The beads were washed three times with the binding buffer and resolved by 2 × SDS loading buffer for SDS-PAGE and western blot analysis.

### Mouse experiments

All mouse experiments were carried out in accordance with the Institutional Animal Care and Use Committee of Nanjing Drum Tower Hospital (SYXK 2014-0052). ICR mice were purchased from the Laboratory Animal Center of Yangzhou University (Yangzhou, China) and were bred at the Laboratory Animal Center of Nanjing Drum Tower Hospital (Nanjing, China). Six-week-old female mice were mated with fertile males to induce pregnancy and 0.5 dpc was considered the day of vaginal plug. In study 1, the uteri were collected on the 1.5, 2.5, 3.5, and 4.5 dpc, and frozen with liquid nitrogen. The samples were used for western blot analysis to investigate protein expression using the indicated antibody. On 1.5 dpc during study 2, 20 μl (2 × 10^8^ TU/side) of Ad-GFP or Ad-GFP-CAPN7 were injected into the uterine lumen. On day 4.5 dpc, the implantation sites were visualized after an intravenous injection of Chicago blue dye. Non-implanted blastocysts were flushed out of the uterus, and the representative image was shown.

### Statistical analysis

Data were analyzed with the unpaired Student’s *t-*test or one-way analysis of variance. Pearson’s correlation analysis was used to assess the relationship between CAPN7 and HOXA10. Each result is shown as the mean ± SD of three independent experiments. *P*-values < 0.05 were considered statistically significant.

## Electronic supplementary material

Supplemental figure 1

Supplemental figure 2

Supplementary Figure Legends
