# Supplementary figures and images for: Calpain7 impairs embryo implantation by downregulating β3-integrin expression via degradation of HOXA10
Source: Cell Death Dis. 2018 Feb 19;9(3):291. doi: 10.1038/s41419-018-0317-3 (PMC5833723; doi:10.1038/s41419-018-0317-3)

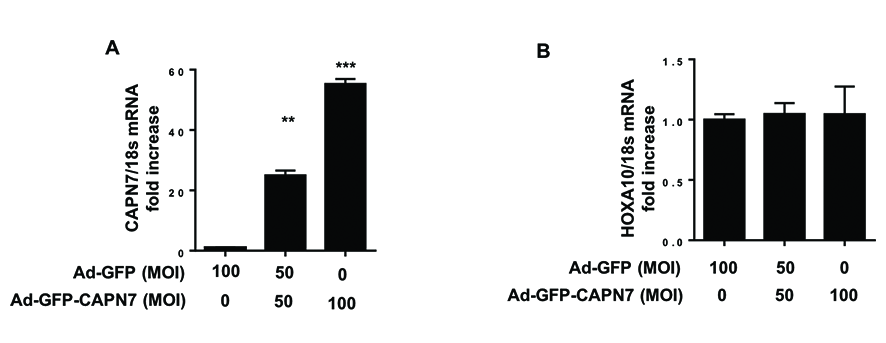

Supplement: Supplementary file 1 — Supplemental figure 1 [file 41419_2018_317_MOESM1_ESM.tif]

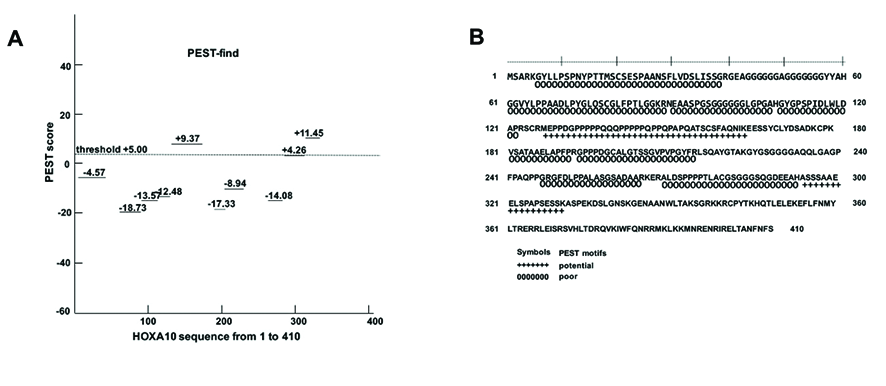

Supplement: Supplementary file 2 — Supplemental figure 2 [file 41419_2018_317_MOESM2_ESM.tif]
