# Supplementary material for: Calpain7 impairs embryo implantation by downregulating β3-integrin expression via degradation of HOXA10
Source: Cell Death Dis. 2018 Feb 19;9(3):291. doi: 10.1038/s41419-018-0317-3 (PMC5833723; doi:10.1038/s41419-018-0317-3)
Supplement: Supplementary file 3 — Supplementary Figure Legends [file 41419_2018_317_MOESM3_ESM.docx]

**Supplementary Figure Legends**

**Figure S1. CAPN7 has no effect on HOXA10 mRNA level**

(A and B) CAPN7 and HOXA10 mRNA expression was measured following CAPN7 overexpression (Ad-GFP-CAPN7, 100 MOI); Values represent the mean ± S.E.M. (n = 3), ***p< 0.01*; ****p<0.001*.

**Figure S2. Schematic representation of PEST motifs in HOXA10**

(A and B ) The PEST score of human HOXA10 is +9.37 and +9.45, as determined by using the computer program *emboss.bioinformatics.nl* . +, PEST potential motif.
